# Adherence to Mediterranean diet impacts gastrointestinal microbial diversity throughout pregnancy

**DOI:** 10.1186/s12884-021-04033-8

**Published:** 2021-08-16

**Authors:** Corrie B. Miller, Paula Benny, Jonathan Riel, Carol Boushey, Rafael Perez, Vedbar Khadka, Yujia Qin, Alika K. Maunakea, Men-Jean Lee

**Affiliations:** 1 John A Burns School of Medicine, University of Hawai’i at Mānoa, Honolulu, USA; 2 John A. Burns School of Medicine, Department of Obstetrics, Gynecology and Women’s Health, 1319 Punahou Street, Suite 824, Honolulu, HI 96826 USA; 3 University of Hawai’i Cancer Center, Epidemiology Program, 701 Ilalo Street Room 525, Honolulu, HI 96813 USA; 4 Epigenomics Research Program, BSB-222K (office)/BSB-228 (lab), 651 Ilalo Street, Honolulu, HI 96813 USA; 5 John A. Burns School of Medicine Department of Quantitative Health Sciences, 651 Ilalo Street, Medical Education Building Suite 411, Honolulu, HI 96813 USA

**Keywords:** Gastrointestinal microbiome, Mediterranean diet, Pregnancy microbiome

## Abstract

**Background:**

Consumption of a diet with high adherence to a Mediterranean diet pattern (MDP) has been associated with a favorable gastrointestinal tract (GIT) microbiome. A healthy GIT microbiome in pregnancy, as defined by increased alpha diversity, is associated with lower chance of adverse perinatal outcomes. This study aimed to evaluate the impact of adherence to an MDP on GIT microbial diversity longitudinally throughout pregnancy.

**Methods:**

Adherence to MDP was scored by the Alternate Mediterranean (aMED) Diet Quality Score, after being applied to a validated Food Frequency Questionnaire. Association of aMED Scores with GIT alpha diversity profiles were compared linearly and across time using a linear mixed model, including covariates of age, body mass index (BMI), ethnicity, and parity.

**Results:**

Forty-one participants of Filipino, Japanese, Native Hawaiian, and Non-Hispanic White descent provided dietary information and microbiome samples during each trimester of pregnancy. Alpha diversity profiles changed over gestation, with decreased microbial diversity in the third trimester. aMED scores positively correlated with Chao1 Index and Observed Species Number (r = 0.244, *p* = 0.017, and r = 0.233, *p* = 0.023, respectively). The strongest association was detected in the third trimester (Chao 1: r = 0.43, *p* = 0.020, Observed Species Number: r = 0.41, *p* = 0.026). Participants with higher aMED scores had higher relative abundance of *Acidaminoacaeae* at the family level (*p* = 0.0169), as well as higher abundance of several species known to increase production of short chain fatty acids within the GIT.

**Conclusions:**

Adherence to MDP pattern is associated with increased maternal GIT microbial diversity, and promotes the abundance of bacteria that produce short chain fatty acids. Increased consumption of fruits, vegetables and legumes with low red meat consumption were key components driving this association. The effect of nutrition however, was less of an effect than pregnancy itself. Further studies are needed to determine if adherence to a Mediterranean diet translates not only into microbial health, but also into reduced risk of adverse pregnancy outcomes.

**Supplementary Information:**

The online version contains supplementary material available at 10.1186/s12884-021-04033-8.

## Background

Gastrointestinal (GIT) microbiota play a role in protecting or promoting adverse pregnancy outcomes. GIT microbial dysbiosis, as defined as low alpha diversity and reduced levels of butyrate producing bacteria, is associated with bacterial translocation and the promotion of endotoxins, creating systemic inflammation. Such inflammation may lead to preterm labor [1], higher blood pressure [2], and increase the risk of developing gestational diabetes [3]. Thus, improving GIT microbial health may help to mitigate adverse pregnancy outcomes.

At baseline, a normal GIT microbial community shifts throughout pregnancy. There is a natural decline in butyrate-producing bacteria from the Firmicutes (*Coprococci, Eubacterium, Roseburia,* and *Faecalibacterium* genera) and *Bacteroidetes* (*Odoribacter* and *Alistipes* genera) phyla [2] at the end of gestation. *Bifidobacteria*, *Proteobacteria*, and lactic acid–producing bacteria increase during the third trimester. This process is thought to help facilitate the normally observed increase in inflammation and weight gain to increase energy supply for the fetus [4]. The end result is less alpha diversity and Operational Taxonomic Unit (OTU) richness by the end of the third trimester. Several studies demonstrate that at this point, the composition resembles that of an individual with metabolic syndrome, with increased *Actinobacteria* and *Proteobacteria*, and decreased *Faecalibacterium* [4, 5]. The rate at which these changes occur and the degree to which alpha diversity changes may play a role in avoiding preterm birth, preeclampsia, or other adverse pregnancy outcomes. Furthermore, manipulating microbial diversity over time may be possible with targeting maternal diet.

Several diet patterns have been associated with improved GIT microbial profiles. Vegetarian diets promote higher levels of *roseburia* and *lacnospiraceae*, and reduce *collinsella*, which is associated with higher levels of circulating insulin [6] [7]. Proportions of high fiber and low-fat intake are correlated with greater microbial diversity and lower levels of *bacteriodaceae* [8]. Polyunsaturated fatty acids also promote gut mucosal integrity and insulin sensitivity by inhibiting inflammation through byproduct fermentation [9]. Particular interest lies in the Mediterranean diet pattern (MDP), which is characterized by high amounts of fiber, lean proteins, fruits and vegetables, and lower consumption of red meats and processed foods. Several studies have noted a beneficial association with consumption of an MDP and GIT microbiome characteristics. MDP adherence is associated with lower levels of lower *Escherichia coli*, higher amounts of *bifidobacteria*, and greater amount of bacterial richness. Such a composition leads to high levels of fecal short-chain fatty acids that contribute to epithelial connections, reduce bacterial translocation and improve systemic inflammation which would otherwise lead to chronic disease [10–13].

Mediterranean diet patterns are also associated with improved maternal and neonatal outcomes including a lower chance of diabetes [14], hypertension during pregnancy [15], excessive gestational weight gain, low birth weight neonates [16], and beneficial metabolic profiling in offspring [17] [18]. The impact of an MDP has not been thoroughly evaluated on the gastrointestinal microbiome during pregnancy. The relationship of improved metabolic health with a Mediterranean diet via increased GIT microbial diversity is established in non-pregnant populations [8] [19], but gaps in knowledge of this relationship among parous women remain. To better understand this relationship, we tested the hypothesis that better adherence to an MDP is associated with higher microbial diversity during pregnancy.

## Methods

### Study subjects and recruitment

This longitudinal cohort study was approved by the Western Institutional Review Board in compliance with Hawai’i Pacific Health protocol. Women were recruited from the 4 most common ethnic groups in Hawai’i – Japanese, Filipino, Native Hawaiian, and non-Hispanic White [20], in the outpatient setting while awaiting first trimester ultrasound appointments. Inclusion criteria were as such: women aged 18–45 years old, primarily English Speaking and English literate, self-identified as Asian, Non-Hispanic White, or Native Hawaiian on intake registration information form and in their first trimester of pregnancy (< 14 weeks 0 days gestation). While many individuals in Hawai’i have a multiethnic background, participants had to identify as 50% or greater (having one parent that is 100% of their reported heritage) to participate in the study. Native Hawaiians of any percent ethnicity were eligible for participation. Participants that identified as 50% one ethnicity and 50% of another ethnicity were excluded.

Other exclusion criteria included: plans to move out of the area prior to delivery, plan to deliver at another hospital other than our medical center, multiple gestation, pre-existing diabetes or hypertension, heart disease, chronic renal disease, systemic lupus erythematosus, hypothyroidism, history of bariatric surgery, history of an eating disorder, or inflammatory bowel disease, and women who are currently incarcerated.

### Data collection

Participation included completing the Multiethnic Cohort Food Frequency Questionnaire (MEC FFQ) three times: once during each trimester, and also collecting microbiome samples via rectal swab at the same time points in each trimester. The first FFQ and bacterial swab collection was completed at time of enrollment, around 11–13 weeks’ gestation. The second collection occurred in the second trimester at the time of their anatomy ultrasound at 18–20 weeks’ gestation. Third trimester samples were collected from 34 to 36 weeks’ gestation.

The MEC-FFQ was developed and validated in a large healthy adult population from 1993 to 1996 in Hawai’i and California [21]. Participants were followed for decades and the tool has proven effective in associating diet with oncologic outcomes and cardiovascular risk. The FFQ includes 182 specific food items uniquely associated with the contemporary local diet such as poi, taro, spam, tofu, salted fish, miso soup, saimin, and fermented foods presumably high in probiotics. Participants were asked recall a typical diet for the previous month. The data extracted from the MEC FFQ was analyzed by the University of Hawai’i Cancer Center Nutrition Shared Support Resource. Adherence to an MDP was scored via the Alternate Mediterranean Diet (aMED) score (Components and Scoring System displayed in Table 1**)** [22]. The analysis was performed on each questionnaire that was completed, so that each participant had up to three scores.

**Table 1:** Scoring Components for Alternate Mediterranean Diet (aMED) Score Adapted from Fung et al., 2005^22^

	Alternate Mediterranean Diet Score	
**Food group**	**Foods included**	**Criteria for 1 point**^*1*^
Vegetables	All vegetables except potatoes	Greater than median intake (servings/d)
Legumes	Tofu, string beans, peas, beans	Greater than median intake (servings/d)
Fruit	All fruit and juices	Greater than median intake (servings/d)
Nuts	Nuts, peanut butter	Greater than median intake (servings/d)
Whole grains	Whole-grain ready-to-eat cereals, cooked cereals, crackers, dark breads, brown rice, other grains, wheat germ, bran, popcorn	Greater than median intake (servings/d)
Red and processed meats	Hot dogs, deli meat, bacon, hamburger, beef	Less than median intake (servings/d)
Fish	Fish and shrimp, breaded fish	Greater than median intake (servings/d)
Ratio of monounsaturated to saturated fat	–	Greater than median intake (servings/d)
Ethanol	Wine, beer, “light” beer, liquor	5–25 g/d

1–0 points if these criteria are not met

### Microbiome sequencing

DNA isolation was performed using the AllPrep DNA/RNA Extraction Kit (Qiagen). ThermoFisher Scientific 16S rRNA primers were then used to create bacterial DNA libraries for sequencing according to manufacturer’s instructions. Metagenomic sequencing was carried out on the Ion Genestudio S5 Sequencer (ThermoFisher Scientific). V2–4-8, V3–6, V7–9 primers are used to amplify the hypervariable regions of the 16S rRNA gene from bacteria. The Ion Torrent Data analysis platform was used to align sequence fragments and provide OTUs at the family, genus, and species level. These reads were used to assign alpha and beta diversity scores through the Ion Torrent Software. The α-diversity indexes were computed after rarefraction was performed, using the average value of the 10 rarefied value at sequence number 15,927.

### Data analysis

Characteristics of the participants were summarized by mean and standard deviation for continuous variable, frequencies, and percentages for categorical variables. Two-tailed Student’s t test, ANOVA or χ^2^ test were used to test the differences of these variables respectively. Variables were log transformed to improve normality and homoscedasticity where appropriate. Non-parametric tests (Mann Whitney U, Kruskal Wallis Test) was applied for non-normally distributed data. Repeated Measures ANOVA with multiple comparison test via Tukey HSD as post-hoc analysis was used to compare alpha diversity (Shannon Index, Chao 1 Scores, Simpson and Observed number of Species) indexes with demographic characteristics such as ethnicity and obesity aggregately among trimesters. Beta diversity profiles were analyzed with PCA among each ethnic group during each trimester after Euclidean Distance Matrix was developed. The primary outcome measures of correlation of aMED score with alpha diversity score were compared with Pearson correlation as well as linear mixed model while accounting for confounders such as age, Body Mass Index (BMI), ethnicity and parity. All data analyses were performed using R Studio version 1.0.136 (http://www.r-project.org/) and a two-tailed *p*-value of less than 0.05 was be regarded as statistically significant.

## Results

For this study, forty-one participants in total were recruited. There were 10 (24.3%) participants each of Non-Hispanic White, Filipino, Japanese descent and 11 (26.8%) participants of Native Hawaiian descent. The average age of the cohort was 29, and the majority were nulliparous (*n* = 22, 56%). Average BMI was 27.2 kg/m2, with only 17% (*n* = 11) being obese. Demographic data for those scoring below versus above the median aMED score are listed in Table 2.

**Table 2:** Composite results from all three trimesters for all participants who completed FFQs, according to Energy adjusted aMED Score (those who scored below and above the median). Aggregate Nutrient Consumption is displayed as mean (SD), and compared via t-test

Composite Energy Adjusted aMED Score	Low (*n* = 21)	High (*n* = 19)	p-value
***Range*:**	[0–4.0]	[4.3–7.0]	
**Age**			
Median [Min, Max]	26.0 [19.0, 38.0]	33.0 [24.0, 40.0]	0.030
**Obesity**			0.978
Normal	10 (47.6%)	9 (47.3%)	
Overweight	5 (23.8%)	5 (26.3%)	
Obese	6 (28.6%)	5 (26.3%)	
**Ethnicity**			0.218
Filipino	8 (38.1%)	2 (10.5%)	
Japanese	4 (19%)	5 (26.3%)	
Native Hawai’i an	4 (19%)	7 (36.8%)	
Non-Hispanic White	5 (23.8%)	5 (26.3%)	
**Parity**			0.672
Nulliparous	11 (52.4%)	11 (57.9%)	
Primaparous	8 (38.1%)	5 (26.3%)	
Multiparous	2 (9.5%)	3 (15.7%)	
**Pregnancy Outcomes**	(available for *n* = 18 in each group)		
Excess Gestational Weight Gain	2	7	
Gestational Diabetes	2	2	
PreEclampsia	5	4	
Spontaneous Preterm Birth	1	0	
Infant Birth weight (grams, Mean [SD])	3110 [507]	3500 [360]	**0.016**
Gestational Age at Delivery (Weeks)	39	38.6	0.762
**Composite Nutritional Components**			
Total Energy (kcal)	2143 (2017)	2181 (1102)	0.942
% carbohydrates from total energy	46.8 (4.6)	50.6 (4)	**0.008**
% protein from total energy	16.3 (2)	15.8 (2)	0.440
% fat from total energy	36.8 (3.4)	33.5 (2.9)	**0.002**
Monounsaturated fat (g)	34.53 (32.4)	31.39 (16)	0.697
Polyunsaturated fat (g)	16.3 (14.4)	17.2 (9.3)	0.794
Cholesterol (mg)	325.8 (314)	295.9 (179)	0.711
Sodium (mg)	3536.4 (2827)	3653.3 (1696.4)	0.874
Fiber (g)	15.6 (13.6)	28.3 (16)	**0.011**
Calcium (mg)	816.5 (807)	997.4 (482)	0.391
Folate (Mg)	447.8 (372)	700.4 (390)	**0.044**
Iron (mg)	13.7 (10.9)	19 (9.9)	0.118

FFQs results were available for 40 participants during the first trimester, 37 for the second trimester, and 33 for the third trimester. Loss to follow-up or withdrawal from the study occurred for 1 patient after the first trimester (termination of pregnancy), and 4 participants after the second trimester collection (1 termination due to pre-viable preeclampsia with severe features, 1 s trimester loss, 1 elective termination and 1 participant moved away). During the third trimester, there were an additional 8 participants who were lost to follow up and did not fill out their survey in the mail, with results for 33 participants in the third trimester. Attempt was made to contact all patients after delivery, regardless if 3rd trimester sample was not collected at 34–36 weeks gestation, in order to gain information about pregnancy outcomes. Pregnancy outcome data was available for 39 of 41 participants. After filtering for DNA quality and samples that yielded greater than 10,000 unique 16S-based sequencing reads, results were available for 35 participants from the first, 36 from the second, and 30 from the third trimester. Data is displayed from participants who had paired data of both FFQ and microbiome in each trimester.

### Diet quality

**Fig. 1:**
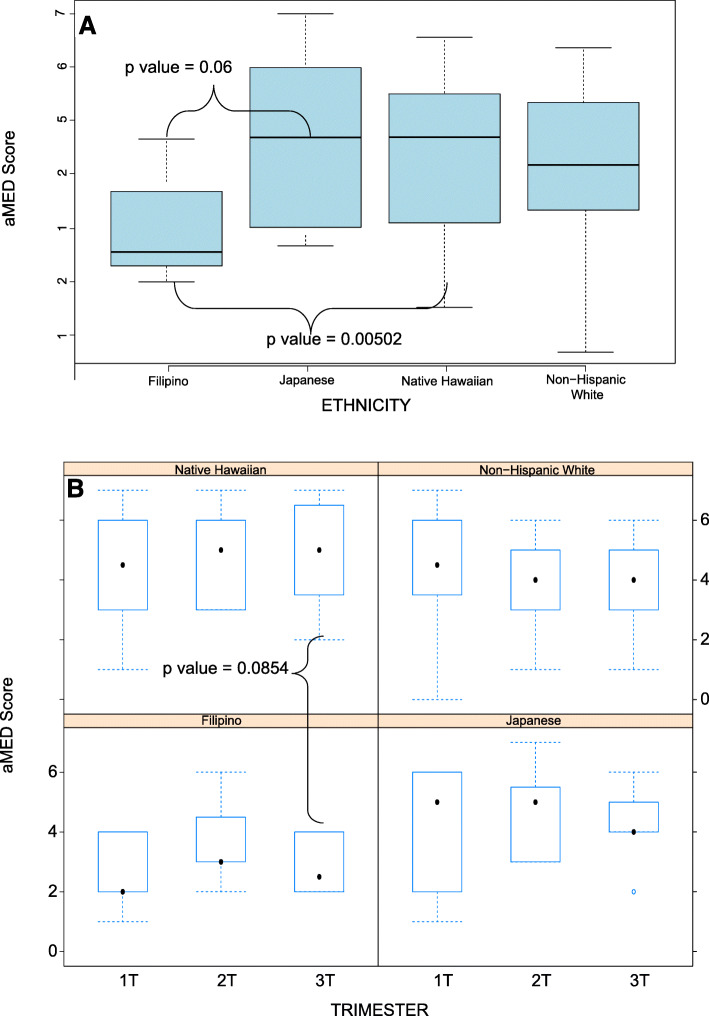
Box plot of aMED scores for each ethnic group aggregately for all trimesters (PANEL A) (mean – solid line, SD- whisker). aMED scores in to each trimester (PANEL B) showing the mean (•) and Standard deviations (dashed lines)

The aggregate distribution of the aMED scores was normally distributed among all three trimesters, (Supplementary Fig. 1). The diet scores among each ethnic group are shown in Fig. 1 – aggregately and according to Trimester (Panel A). Overall, Native Hawaiian participants had higher adherence to Mediterranean diet quality than Filipino participants (*p* = 0.005), as did Japanese (non-significant (NS), *p* = 0.06). These differences were primarily comprised of scores in the third trimester, as demonstrated in Fig. 1 Panel B (NS). There were no differences in scores of those who are obese versus non-obese (mean = 3.9 vs 4.2, *p* = 0.56).

### Microbiome data

**Fig. 2:**
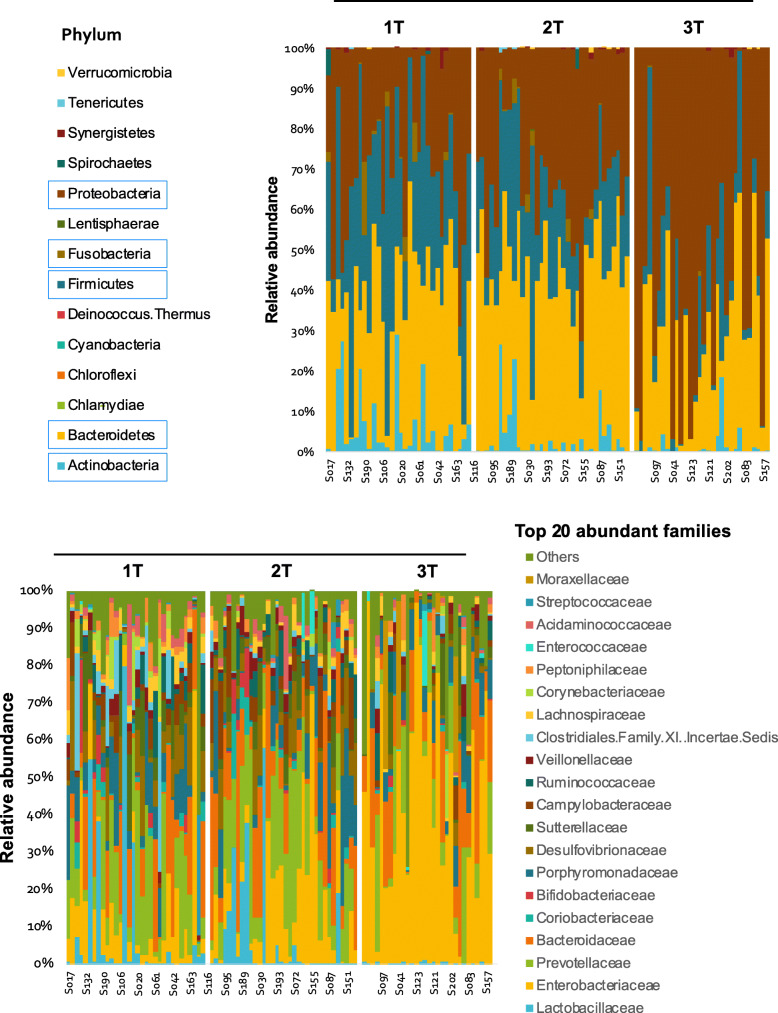
Aggregate OTU abundance at the family level across all trimesters according to ethnicity

Overall, 577 different OTUs were identified. Differences were detected among aggregate abundance at the family level among ethnicities. We observed greater abundance of *lactobacillaceae* in Japanese and Filipino compared to Non-Hispanic White and Native Hawaiian participants (*p* = 0.018). Non-Hispanic White women tended to have higher *Porphyromonadaceae* (NS). Native Hawaiians had higher levels of *Acidaminococcaceae* (NS) (Fig. 2), and the highest ratios of *Prevotellaceae* to *Bacteroidaceae*, versus Non-Hispanic white women had the lowest ratios (NS).

**Fig. 3:**
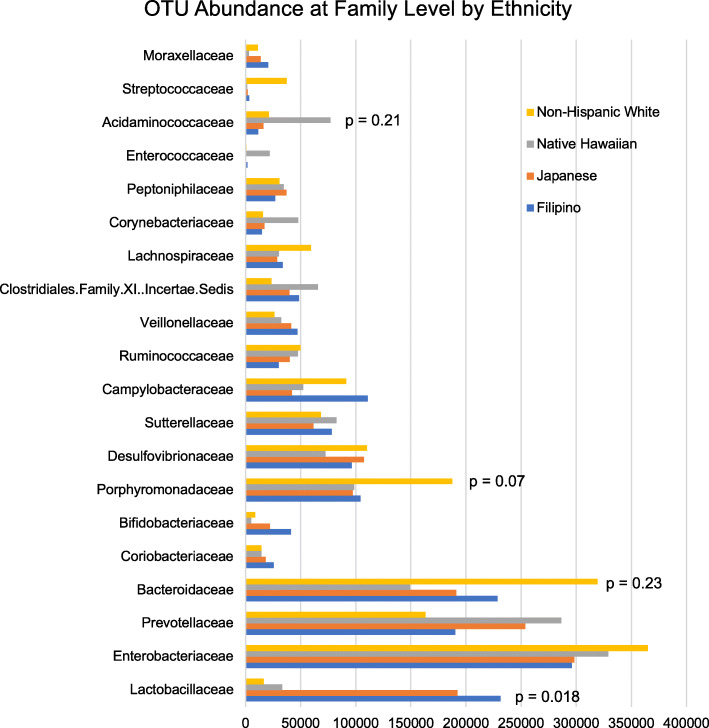
Phylum (Top panel) and Family (bottom panel) distribution across all samples according to trimester. The most abundant phyla are *Proteobacteria*, *Fusobacteria, Firmicutes, Bacteroidetes,* and *Actinobacteria*

**Fig. 4:**
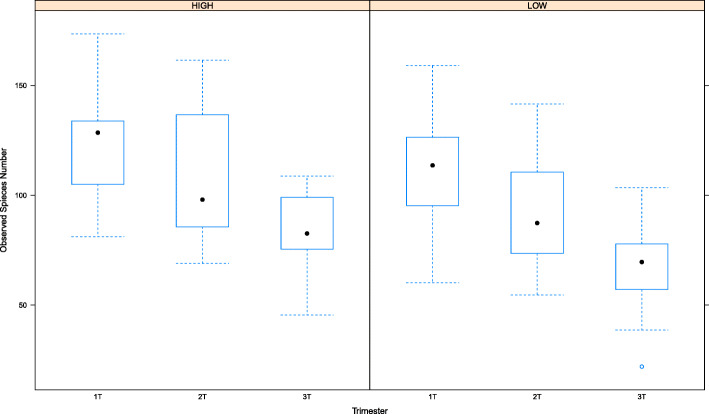
Alpha Diversity profiles according to trimester for those averaging above and below the median aMED diet quality score

**Fig. 5:**
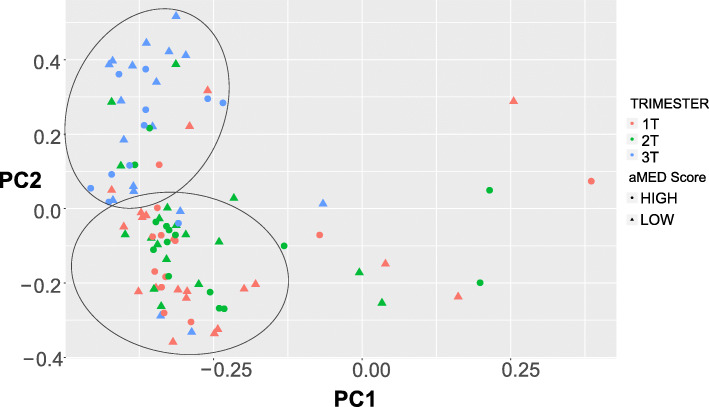
Principal Component Analysis of OTUs at the family level, compared by aMED Score and Trimester. aMED Scores above the mean are represented by circles and scores below the mean are represented by triangles

The most abundant phyla and families according to trimester are shown in Fig. 3. There is a shift in the types and abundance of microbes present between the first and second trimesters to the third trimester. Consistent with other observations [4], alpha diversity decreased significantly over time from first to third trimester. The rate of change was not different between those who scored above and below the mean aMED Score (Fig. 4). There was also no difference detected in the rate of change over time among participants of different ethnicities or those who were obese versus non-obese (not shown). Principal Component Analysis plots are shown to compare samples. Third trimester samples segregate away from first and second in the distance matrix (Fig. 5), demonstrating a shift of microbial composition at the end of gestation. There was no distinct grouping among those who had higher aMED diet quality scores versus those who had lower scores, and no distinct groupings among ethnicity or BMI class (not shown).

**Fig. 6:**
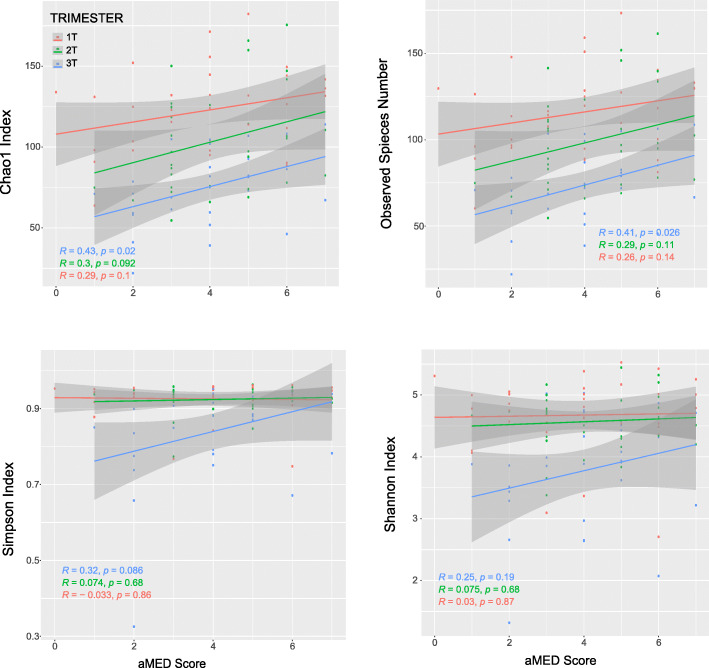
Pearson correlation of each alpha diversity metric with aMED Score according to Trimester. Correlation Coefficients (R) are listed in order of Trimester (1st, 2nd, and 3rd), with associated *p*-values

The primary test for a relationship between alpha diversity metrics and aMED Score was evaluated as a linear correlation, with the hypothesis that better adherence to the Mediterranean diet would increase alpha diversity. While bivariate analysis of those above and below the mean did not show a rate of change over time (Fig. 4), there was linear correlation with aMED score and alpha diversity of the GIT microbiome. Table 3 shows the correlation coefficient for all diversity metrics with combined aMED scores, irrespective of trimester. The correlation is primarily supported by the association in the third trimester with regards to overall richness, as measured by Chao1 Index and Observed species number (Fig. 6). Specifically, this comparison is visualized in a scatterplot, with aMED score on the X axis, and alpha diversity measures on the Y-axis (Fig. 6). Overall evenness of the species (as measured by Shannon and Simpson index) was not impacted by aMED score (Table 2 and Fig. 6). To determine the relationship between observed species number and aMED score, multivariate linear mixed-effect model was implemented using lme4 package in R. Fixed effect covariates included obesity, parity, ethnicity, trimester and age, while subject was treated as random effects. Visual inspection of residual plots did not reveal any obvious deviations from homoscedasticity or normality. No significant correlation or covariate was produced from the model, except for aMED score (B-estimate 4.83, 95% Confidence Interval 1.5–8.14).

**Table 3:** Correlation between aggregate aMED score and α-diversity indexes

Diversity index	r	p
**Chao1**	0.244	0.017
**Observed Species #**	0.233	0.023
**Shannon**	0.103	0.321
**Simpson**	0.137	0.1862

To further understand the relationship above, the OTU abundance at the family level was compared via non-parametric t-test among those with high versus low aMED scores. There is a greater abundance of *Acidaminoaceae* in the those that scored the highest compared to than those with the lowest aMED scores. (*p* = 0.0169). Interestingly, there is also a trend for higher diet quality being associated with increased *Prevotellaceae*. The ratio of *Prevotellaceae* to *Bacteroidaceae* was normalized by log transformation and compared among high and low aMED scores. Those with higher aMED scores had higher *Prevotellaceae: Bacteroidaceae* ratios (**Supplementary Material** Fig. 2), especially in the third trimester. At the species level, several species associated with the highest AMED scores (> 6) and are listed in Table 4.

**Table 4:** Species with significant correlations with the highest aMED Scores (> 6)

Genus_Species	Correlation (r)	***p***-value	Metabolic Production and Physiologic Effects cited in literature
Lactobacillus_rogosae	0.277	0.007	Decreased in women with Gestational Diabetes [23]
Coprococcus_eutactus	0.256	0.012	Actively ferments carbohydrates, produces butyric and acetic acids with formic or propionic and/or lactic acid [24]
Phascolarctobacterium_faecium	0.243	0.018	Produces short-chain fatty acids, including acetate and propionate, upregulated by metformin in animal models [25]
Anaerococcus_tetradius	0.242	0.018	Primarily found in vagina and female reproductive tract. Ferments glucose and mannose, butyrate is an end-product [26]
Collinsella_aerofaciens	0.235	0.022	Unique *collinsella* species with butyrate kinase [27]
Alistipes_sp.	0.229	0.025	Negative correlation with glucose intolerance in pregnancy [28]
Faecalibacterium_prausnitzii	0.205	0.046	Butyrate producing, Anti-inflammatory properties, associated with low secretion of pro-inflammatory cytokines (IL-12 and IFN-γ), and elevated secretion of the anti-inflammatory cytokine IL-10 [36, 38, 39].
Pseudobutyrivibrio_ruminis	0.202	0.050	Butyrate producer [23]

**Fig. 7:**
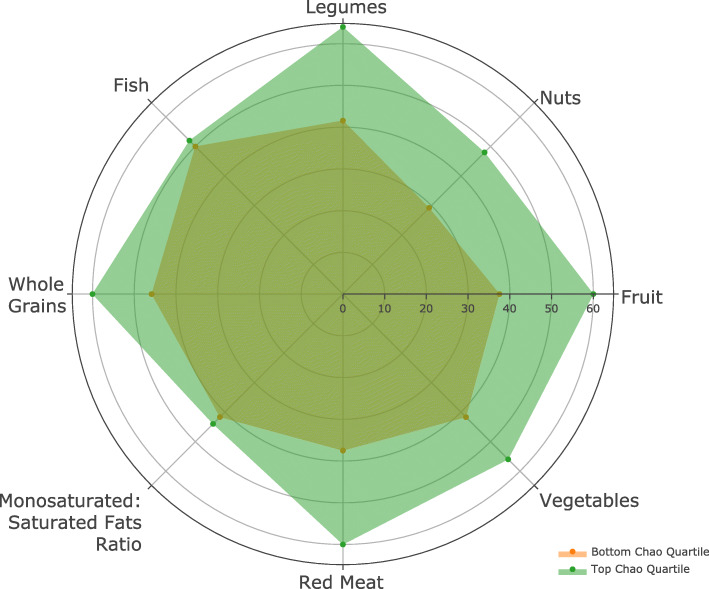
Components of aMED Score for paired GIT microbiome samples with the lowest and highest alpha diversity as measured by specimens in the bottom and top 25th percentiles of Chao1 Index scores

Fig. 7 shows the differences in components of the aMED score in those with the highest (green) and lowest (orange) Chao1 scores. Participants with greater alpha diversity as measured by the Chao1 Index tended score a point more frequently in the aMED categories of vegetables, fruit, nuts, legumes, and red meat (equating to less red meat), compared to those with lower Chao1 scores. There were not large differences in the amount of fish or monosaturated to saturated fats consumed. Other macro and micronutrients did not differ between those of high versus low alpha diversity scores, except for the amount of polyunsaturated fats. And while those with higher aMED scores consumed larger amounts of fiber, this was not independently correlated with alpha diversity metrics. There were no differences in the amount of fiber between those of high versus low Chao1 scores.

### Pregnancy outcomes

Pregnancy data was available for 39 participants from the cohort, and complete paired data for 36 participants. Nine (25%) women developed Pregnancy-induced Hypertension (PIH) (including gestational hypertension, PreEclampsia with and without severe features). Four participants (11.11%) developed gestational diabetes (GDM), and 10 (27.8%) participants had excess gestational weight gain (GWG) beyond the amount of gestational weight gain recommended by the Institute of Medicine (IOM). One participant had a second trimester loss due to cervical insufficiency, and another preterm birth at 36 weeks. Exploratory analysis was performed to look at mean differences of alpha diversity profiles among women who did and did not develop the most common adverse pregnancy outcomes (PIH, GDM, and excess GWG). **(Supplementary Material** Table 1**).** Comparisons were made from mean scores across all trimesters and according to trimester. No consistent patterns were demonstrated for any of the adverse pregnancy outcomes. Interestingly, women with excess gestational weight gain had higher aMED scores, which is not consistent with previous studies [15], and reflective of the small sample size. Abundance of particular families or genera were not associated with any of these adverse pregnancy outcomes (not shown).

## Discussion

The relationship of improved metabolic health with a Mediterranean diet via increased GIT microbial diversity during pregnancy is not well understood, in particular among ethnically diverse women. To our knowledge, this study is the first to characterize dietary patterns and GIT microbiome in a multi-ethnic cohort in the Pacific, and examine this relationship at the microbial species level. The unique makeup of Hawai’i residents is infrequently represented in dietary research of pregnant women [29–31]. The population consists of immigrants, first generation individuals, and a large heterogenous group of mixing cultures. As such, dietary patterns are influenced from a variety of sources. There is also a unique consumption of diverse fermented foods such as nato and poi that may contribute to a distinct gut microbiota.

Our cohort followed the widely described transition to decreased diversity under the influence of hormonal shifts across gestation. Commensurate with other published studies of pregnant populations [32] [4], there were significant differences in Shannon, Chao1, Simpson, and observed number of species from first trimester to third trimester. The changes were mostly represented in a decrease in total abundance of *lactobacillaceae* (*p* = 0.013), and *lachnospiracheae* (*p* = 0.00489), and an increase in *prevotellaceae* (*p* = 0.03). On top of this pregnancy shift, MDP adherence influenced microbial diversity over time, even while accounting for BMI, ethnicity and parity. While other studies have demonstrated that diet has more of an influence on microbial composition than body mass index, this was masked in our study by the pregnant cohort [33]. Our primary outcome, a linear correlation with diet quality as measured by adherence to MDP and microbial diversity was demonstrated; Women with higher adherence to the MDP had a smaller decrease in alpha diversity in the third trimester. The benefits of adherence to an MDP is well documented both on development of chronic disease, and likely mechanisms by which these occur – namely inflammation, and aberrant gut microbiota. These relationships have not been specifically explored during pregnancy. Our results offer novel insight into the impact that diet had on microbial composition and richness over pregnancy. Specifically, despite a normal transition to more dysbiosis in the GIT during the third trimester, this shift was mitigated by better adherence to MDP.

One other recent study describes the impact of dietary quality on GIT microbial health during pregnancy. Laitinen et al. measured diet quality by the “Index of Diet Quality” (IDQ) score and alpha diversity in early pregnancy (one time point at less than 18 weeks gestation) [34]. Researchers detected a correlation of diet quality and all indices (Shannon Index, Chao, observed Species number and phylogenetic diversity) within this cohort of overweight and obese women in Finland. They noted the two food components most associated with increased alpha diversity were whole grains and vegetables, similar to our findings. Both our study and Laitinen’s demonstrate that comprehensive diet quality is beneficial for maternal GIT microbial health. While other research has focused on particular micro or macronutrients [8, 35] impact on GIT microbiome, we sought to understand how holistic and balanced nutrition improves the gut microbiome, as this type of dietary pattern is the easiest to which to adhere.

At the species level, several species were noted to be more abundant in those with the highest aMED scores (> 6). While the literature is limited in descriptions of how these organisms directly impact human health, some of the identified species have been described in patients with autoimmune or inflammatory conditions [27, 36, 37]. Several of the species more abundant in participants with higher aMED scores are described as producing beneficial by products such a butyrate and acetic acid (As described in Table 4) [23]. For instance, there was greater abundance of *lactobacillus rogosae* and *alisteps sp.* in women with higher aMED Scores in our cohort. These organisms are observed to be decreased in women with gestational diabetes or glucose intolerance during pregnancy [28, 38]**.** *Coprococcus* and *A. tetradius* are also known to produce butyrate and acetic acid, supporting tight mucosal adhesion within the gastrointestinal tract and thus decreasing systemic inflammation [26, 39]. Likewise, *Faecalibacterium prausnitzii* is associated with decreased cytokine production in patients with Crohn’s disease [25, 40].

Looking at the specific enterotypes represented in this cohort, *Prevotella* was highly abundant in participants with higher diet quality scores [34]. Within the GIT microbiome, a *Prevotella*-dominant enterotype is associated with high intake of fiber, carbohydrate, and simple sugars, whereas *Bacteroides*-dominant enterotype is associated with the high intake of animal fat and protein [24]. De Fillipo et al. and Martinez et al. both describe a *Prevotella* dominant enterotype is prevalent in populations eating a more traditional diet versus Westernized dietary pattern [41] [42]. As such, this composition led to overall greater microbial richness, and produced higher levels of short-chain fatty acids. In our cohort, Native Hawaiians had the highest abundance of *Prevotellaceae*, and also had higher adherence to MDP. Yet, this population also has some of the highest adverse pregnancy outcomes in Hawai’i [43, 44]. Further investigation of these relationships is needed to understand what contribution nutrition plays into ethnic-specific disparate birth outcomes.

Mechanistic knowledge is still needed before the connection to translatable, modifiable health interventions utilizing the microbiome is achieved. This study aimed to provide more baseline knowledge about nutrition and the GIT microbiome in pregnancy with the aim to generate new areas of investigation. The majority of research on the microbiome in pregnancy thus far has been directed toward characterizing vaginal microbial communities, with an emphasis on the association with preterm birth. Many studies demonstrate that increased vaginal microbial diversity and depletion lactobacillus-dominant communities are associated with preterm birth [45–47]. Researchers hypothesize this is due to acquisition of pathogenic organisms, or from metabolites produced by anerobic microbes [48]. Conversely, little is yet known regarding the GIT microbiome in pregnancy. The specific families, species or communities that are most important have yet to be characterized, and interventions such as probiotics to affect pregnancy health have thus far proven futile [49]. Furthermore, the vaginal and GIT microbiome are closely related, and additional studies are needed to demonstrate how both the vaginal and GIT microbial communities, which are interconnected, contribute to adverse pregnancy outcomes, as well as interventions that can be utilized to impact health outcomes.

Limitations of this study include inherent recall bias by using a Food Frequency Questionnaire. However, FFQs are often structured to have patients recall what they eat over a predetermined time period. FFQs have been shown to be as accurate as 24-h recall in correlation with biologic specimens showing metabolites and nutrients [50]. Another limitation is our limited sample size. The authors acknowledge the limitation in the observational nature of this small study as hypothesis-generating. The cohort was not powered to measure the ultimate impact of microbial diversity of adverse pregnancy outcomes. While an attempt was made to account for other confounding factors that influence microbial health, some aspects were not captured such as exercise, maternal adiposity, or domestic environment (rural versus urban). However, this study lays the groundwork for future research for understanding the contributions of GIT microbial dysbiosis, nutrition and prevention of adverse perinatal outcomes, and biologic mechanisms by which this occurs. The strengths of this study include it being the first-time diet quality and microbial composition has been described in pregnant women in Hawai’i. Measuring these relationships and changes longitudinally across all three trimesters makes this insightful information even more impactful.

## Conclusions

Ultimately, microbial diversity is comprised of several environmental factors including adiposity, environment, geography and diet. The strongest determinant observed in our cohort was pregnancy itself, with the hormonal changes leading to an expected decrease in diversity over time. While to a lesser degree, adherence to MDP was also impactful on alpha diversity within the GIT and should be considered when discussing nutrition recommendations with pregnant women.

## Supplementary Information

**Additional file 1: Table S1.** Mean values [with (Standard Deviation) below] of alpha diversity and aMED scores were compared among those who did and did not develop the most common pregnancy complications in the cohort. Pregnancy outcome and microbiome data was available for 36 participants.

**Additional file 2: Fig. S1.** Aggregate distribution of all reported energy adjusted aMED scores.

**Additional file 3: Fig. S2.***Prevotella:Bacteroides* Ratios among those above (right panel) and below (left panel) the median aMED score. Participants with lower aMED scores had lower ratios in the third trimester.

## Data Availability

Following the acceptance of our manuscript for publication, these datasets will be deposited into appropriate databases including the NCBI Gene Expression Omnibus (GEO) database, the NCBI Short Read Archives (SRA), MicrobiomeDB, and other relevant databases and made freely available to investigators at academic institutions worldwide.

## Abbreviations

aMED: Alternate Mediterranean Diet
BMI: Body Mass Index
GDM: Gestational Diabetes
GIT: Gastrointestinal
GWG: Gestational Weight Gain
IDQ: Index of Diet Quality
IOM: Institute of Medicine
MDP: Mediterranean diet pattern
MEC FFQ: Multiethnic Cohort Food Frequency Questionnaire
OTU: Operational Taxonomic Unit
PIH: Pregnancy-Induced Hypertension

## References

[1] Dahl C, Stanislawski M, Iszatt N, Mandal S, Lozupone C, Clemente JC, Knight R, Stigum H, Eggesbo M (2017). Gut microbiome of mothers delivering prematurely shows reduced diversity and lower relative abundance of Bifidobacterium and Streptococcus. PLoS One.

[2] Gomez-Arango LF, Barrett HL, McIntyre HD, Callaway LK, Morrison M, Dekker Nitert M, Group ST (2016). Increased systolic and diastolic blood pressure is associated with altered gut microbiota composition and butyrate production in early pregnancy. Hypertension.

[3] Crusell MKW, Hansen TH, Nielsen T, Allin KH, Ruhlemann MC, Damm P, Vestergaard H, Rorbye C, Jorgensen NR, Christiansen OB (2018). Gestational diabetes is associated with change in the gut microbiota composition in third trimester of pregnancy and postpartum. Microbiome.

[4] Koren O, Goodrich JK, Cullender TC, Spor A, Laitinen K, Backhed HK, Gonzalez A, Werner JJ, Angenent LT, Knight R (2012). Host remodeling of the gut microbiome and metabolic changes during pregnancy. Cell.

[5] Liu J, Yang H, Yin Z, Jiang X, Zhong H, Qiu D, Zhu F, Li R (2017). Remodeling of the gut microbiota and structural shifts in preeclampsia patients in South China. Eur J Clin Microbiol Infect Dis.

[6] Gomez-Arango LF, Barrett HL, Wilkinson SA, Callaway LK, McIntyre HD, Morrison M, Dekker Nitert M (2018). Low dietary fiber intake increases Collinsella abundance in the gut microbiota of overweight and obese pregnant women. Gut Microbes.

[7] Barrett HL, Gomez-Arango LF, Wilkinson SA, McIntyre HD, Callaway LK, Morrison M, et al. A vegetarian diet is a majordeterminant of gut microbiota composition in early pregnancy. Nutrients. 2018;10(7). 10.3390/nu10070890. 10.3390/nu10070890 PMC6073691 30002323

[8] Roytio H, Mokkala K, Vahlberg T, Laitinen K (2017). Dietary intake of fat and fibre according to reference values relates to higher gut microbiota richness in overweight pregnant women. Br J Nutr.

[9] Alcock J, Lin HC (2015). Fatty acids from diet and microbiota regulate energy metabolism. F1000Res.

[10] Gutierrez-Diaz IF-NT, Salazar N, Bartolome B, Moreno-Arribas MV, de Andres-Galiana EJ, Fernandez-Martinez JL, Reyes-Gavilan CGD, Gueirnonde M, Gonzalez S (2017). Adherence to a Mediterranean diet influences the fecal metabolic profile of microbial- derived phenolics in a Spanish cohort of middle-age and older people. J Agric Food Chem.

[11] Mitsou EK, Kakali A, Antonopoulou S, Mountzouris KC, Yannakoulia M, Panagiotakos DB, Kyriacou A (2017). Adherence to the Mediterranean diet is associated with the gut microbiota pattern and gastrointestinal characteristics in an adult population. Br J Nutr.

[12] Garcia-Mantrana I, Selma-Royo M, Alcantara C, Collado MC (2018). Shifts on gut microbiota associated to Mediterranean diet adherence and specific dietary intakes on general adult population. Front Microbiol.

[13] De Filippis FPN, Vannini L, Jeffery IB, La Storia A, Laghi L, Serrazanetti DI, Di Cagno R, Ferrocino I, Lazzi C (2016). High-level adherence to a Mediterranean diet beneficially impacts the gut microbiota and associated metabolome. Gut.

[14] Amati F, Hassounah S, Swaka A. The impact of Mediterranean dietary patterns during pregnancy on maternal and offspringhealth. Nutrients. 2019;11(5). 10.3390/nu11051098. 10.3390/nu11051098 PMC6566342 31108910

[15] Schoenaker DA, Soedamah-Muthu SS, Mishra GD (2016). Quantifying the mediating effect of body mass index on the relation between a Mediterranean diet and development of maternal pregnancy complications: the Australian longitudinal study on Women's health. Am J Clin Nutr.

[16] Martinez-Galiano JM, Olmedo-Requena R, Barrios-Rodriguez R, Amezcua-Prieto C, Bueno-Cavanillas A, Salcedo-Bellido I, et al. Effect of adherence to a Mediterranean diet and olive oil intake during pregnancy on risk of small for gestational age infants.Nutrients. 2018;10(9). 10.3390/nu10091234. 10.3390/nu10091234 PMC6164545 30189597

[17] Gesteiro ERBB, Bastida S, Sánchez-Muniz FJ (2012). Maternal diets with low healthy eating index or Mediterranean diet adherence scores are associated with high cord-blood insulin levels and insulin resistance markers at birth. Eur J Clin Nutr.

[18] Lorite Mingot D, Gesteiro E, Bastida S, Sanchez-Muniz FJ (2017). Epigenetic effects of the pregnancy Mediterranean diet adherence on the offspring metabolic syndrome markers. J Physiol Biochem.

[19] Maskarinec G, Hullar MAJ, Monroe KR, Shepherd JA, Hunt J, Randolph TW, Wilkens LR, Boushey CJ, Le Marchand L, Lim U, Lampe JW (2019). Fecal microbial diversity and structure are associated with diet quality in the multiethnic cohort adiposity phenotype study. J Nutr.

[20] U.S. Census Bureau: 2012-2016 American Community Survey 5-Year Estimates. https://www.census.gov. Accessed 18 Oct 2018.

[21] Kolonel LN, Henderson BE, Hankin JH, Nomura AM, Wilkens LR, Pike MC, Stram DO, Monroe KR, Earle ME, Nagamine FS (2000). A multiethnic cohort in Hawaii and Los Angeles: baseline characteristics. Am J Epidemiol.

[22] Fung TTMM, Newby PK, Manson JE, Meigs JB, Rifai N, Willett WC, Hu FB (2005). Diet-quality scores and plasma concentrations of markers of inflammation and endothelial dysfunction. Am J Clin Nutr.

[23] Van Gylswyk NO, Hippe H, Rainey FA (1996). *Pseudobutyrivibrio ruminis* gen. nov., sp. nov., a Butyrate-Producing Bacterium from the Rumen That Closely Resembles *Butyrivibrio fibrisolvens* in Phenotype. Int J Syst Bacteriol.

[24] Wu GD, Chen J, Hoffmann C, Bittinger K, Chen YY, Keilbaugh SA, Bewtra M, Knights D, Walters WA, Knight R (2011). Linking long- term dietary patterns with gut microbial enterotypes. Science.

[25] Sokol H, Pigneur B, Watterlot L, Lakhdari O, Bermudez-Humaran LG, Gratadoux JJ, Blugeon S, Bridonneau C, Furet JP, Corthier G, Grangette C, Vasquez N, Pochart P, Trugnan G, Thomas G, Blottiere HM, Dore J, Marteau P, Seksik P, Langella P (2008). Faecalibacterium prausnitzii is an anti-inflammatory commensal bacterium identified by gut microbiota analysis of Crohn disease patients. Proc Natl Acad Sci U S A.

[26] Ezaki T, Kawamura Y, Li N, Li ZY, Zhao L, Shu S (2001). Proposal of the genera Anaerococcus gen. Nov., Peptoniphilus gen. Nov. and Gallicola gen. Nov. for members of the genus Peptostreptococcus. Int J Syst Evol Microbiol.

[27] Qin P, Zou Y, Dai Y, Luo G, Zhang X, Xiao L. Characterization a novel butyric acid-producing bacterium Collinsella aerofaciens Subsp. Shenzhenensis Subsp Nov. Microorganisms. 2019;7:1-10. 10.3390/microorganisms7030078. 10.3390/microorganisms7030078 PMC6463082 30871249

[28] Wu Y, Bible PW, Long S, Ming WK, Ding W, Long Y, Wen X, Li X, Deng X, Deng Y, Guo S, Doçi CL, Wei L, Chen H, Wang Z (2020). Metagenomic analysis reveals gestational diabetes mellitus-related microbial regulators of glucose tolerance. Acta Diabetol.

[29] Rifas-Shiman SL, Rich-Edwards JW, Kleinman KP, Oken E, Gillman MW (2009). Dietary quality during pregnancy varies by maternal characteristics in project viva: a US cohort. J Am Diet Assoc.

[30] Rifas-Shiman SL, Rich-Edwards JW, Willett WC, Kleinman KP, Oken E, Gillman MW (2006). Changes in dietary intake from the first to the second trimester of pregnancy. Paediatr Perinat Epidemiol.

[31] Shapiro AL, Kaar JL, Crume TL, Starling AP, Siega-Riz AM, Ringham BM, Glueck DH, Norris JM, Barbour LA, Friedman JE, Dabelea D (2016). Maternal diet quality in pregnancy and neonatal adiposity: the healthy start study. Int J Obes.

[32] Smid MC, Ricks NM, Panzer A, McCoy AN, Azcarate-Peril MA, Boggess KA, Keku TO (2018). Maternal gut microbiome biodiversity in pregnancy. Am J Perinatol.

[33] Davis SC, Yadav JS, Barrow SD, Robertson BK. Gut microbiome diversity influenced more by the westernized dietary regime than the body mass index as assessed using effect size statistic. Microbiologyopen. 2017;6(4). 10.1002/mbo3.476. 10.1002/mbo3.476 PMC5552927 28677210

[34] Laitinen K, Mokkala K. Overall dietary quality relates to gut microbiota diversity and abundance. Int J Mol Sci. 2019;20(8). 10.3390/ijms20081835. 10.3390/ijms20081835 PMC6515207 31013927

[35] Mokkala K, Roytio H, Munukka E, Pietila S, Ekblad U, Ronnemaa T, Eerola E, Laiho A, Laitinen K (2016). Gut microbiota richness and composition and dietary intake of overweight pregnant women are related to serum Zonulin concentration, a marker for intestinal permeability. J Nutr.

[36] Zhou CZH, Xiao XY, Chen BD, Guo RJ, Wang Q, Chen H, Zhao LD, Zhang CC, Jiao YH, Ju YM, Yang HX, Fei YY, Wang L, Shen M, Li H, Wang XH, Lu X, Yang B, Liu JJ, Li J, Peng LY, Zheng WJ, Zhang CY, Zhou JX, Wu QJ, Yang YJ, Su JM, Shi Q, Wu D, Zhang W, Zhang FC, Jia HJ, Liu DP, Jie ZY, Zhang X (2020). Metagenomic profiling of the pro-inflammatory gut microbiota in ankylosing spondylitis. J Autoimmun.

[37] Kang DWIZ, Isern NG, Hoyt DW, Howsmon DP, Shaffer M, Lozupone CA, Hahn J, Adams JB, Krajmalnik-Brown R (2018). Differences in fecal microbial metabolites and microbiota of children with autism spectrum disorders. Anaerobe.

[38] Festa C, Corleto VD, Toscano M, Bitterman O, Drago L, Napoli A (2018). Flash on gut microbiome in GDM and non-GDM pregnancies. Diabetes.

[39] Holdeman LVM, Moore WE (1974). New Genus, Coprococcus, Twelve New Species, and Emended Descriptions of Four Previously Described Species of Bacteria from Human Feces. Int J Syst Bacteriol.

[40] Lopez-Siles M, Duncan SH, Garcia-Gil LJ, Martinez-Medina M (2017). Faecalibacterium prausnitzii: from microbiology to diagnostics and prognostics. ISME J.

[41] De Filippo CCD, Di Paola M, Ramazzotti M, Poullet JB, Massart S, Collini S, Pieraccini G, Lionetti P (2010). Impact of diet in shaping gut microbiota revealed by a comparative study in children from Europe and rural Africa. Proc Natl Acad Sci U S A.

[42] Horwood PF, Tarantola A, Goarant C, Matsui M, Klement E, Umezaki M, Navarro S, Greenhill AR (2019). Health challenges of the Pacific region: insights from history, geography, social determinants, genetics, and the microbiome. Front Immunol.

[43] March of Dimes Report Card. https://www.marchofdimes.org/peristats/tools/reportcard.aspx?frmodrc=1&reg=15. Accessed 12 Jan 2021.

[44] Chang AL, Hurwitz E, Miyamura J, Kaneshiro B, Sentell T (2015). Maternal risk factors and perinatal outcomes among pacific islander groups in Hawaii: a retrospective cohort study using statewide hospital data. BMC Pregnancy Childbirth.

[45] Hyman RW, Fukushima M, Jiang H, Fung E, Rand L, Johnson B, Vo KC, Caughey AB, Hilton JF, Davis RW, Giudice LC (2014). Diversity of the vaginal microbiome correlates with preterm birth. Reprod Sci.

[46] Brown RG, Marchesi JR, Lee YS, Smith A, Lehne B, Kindinger LM, Terzidou V, Holmes E, Nicholson JK, Bennett PR, MacIntyre DA (2018). Vaginal dysbiosis increases risk of preterm fetal membrane rupture, neonatal sepsis and is exacerbated by erythromycin. BMC Med.

[47] Stout MJ, Zhou Y, Wylie KM, Tarr PI, Macones GA, Tuuli MG (2017). Early pregnancy vaginal microbiome trends and preterm birth. Am J Obstet Gynecol.

[48] Stafford GP, Parker JL, Amabebe E, Kistler J, Reynolds S, Stern V, et al. Spontaneous preterm birth is associated with differential expression of vaginal metabolites by lactobacilli-dominated microflora. Front Physiol. 2017;8. 10.3389/fphys.2017.00615. 10.3389/fphys.2017.00615 PMC5572350 28878691

[49] Callaway LK, McIntyre HD, Barrett HL, Foxcroft K, Tremellen A, Lingwood BE, et al. Probiotics for the prevention of gestational diabetes mellitus in overweight and obese women: findings from the SPRING double-blind randomized controlled trial. Diabetes Care. 2019. p. dc182248. 10.2337/dc18-2248. 10.2337/dc18-2248 30659070

[50] Feskanich DRE, Giovannucci EL, Colditz GA, Stampfer MJ, Litin LB, Willett WC (1993). Reproducibility and validity of food intake measurements from a semiquantitative food frequency questionnaire. J Am Diet Assoc.

