# Supplementary material for: Adherence to Mediterranean diet impacts gastrointestinal microbial diversity throughout pregnancy
Source: BMC Pregnancy Childbirth. 2021 Aug 16;21:558. doi: 10.1186/s12884-021-04033-8 (PMC8369757; doi:10.1186/s12884-021-04033-8)
Supplement: Supplementary file 1 — Additional file 1: Table S1. Mean values [with (Standard Deviation) below] of alpha diversity and aMED scores were compared among those who did and did not develop the most common pregnancy complications in the cohort. Pregnancy outcome and microbiome data was available for 36 participants. [file 12884_2021_4033_MOESM1_ESM.docx]

|  |  | Chao1 | | | Observed Species Number | | | | Shannon | | | | Simpson | | | | | aMED Score | | |
| --- | --- | --- | --- | --- | --- | --- | --- | --- | --- | --- | --- | --- | --- | --- | --- | --- | --- | --- | --- | --- |
|  | Trimester: | No | Yes | p-value | | No | Yes | p-value | | No | Yes | p-value | | No | Yes | p-value | No | | Yes | p-value |
| Pregnancy Induced Hypertension  Yes: n =9  No: n = 27 | Overall | 96.94 (34.62) | 115.27 (30.45) | 0.580 | | 96.92 (31.26) | 93.98 (28.57) | 0.685 | | 4.34 (0.823) | 4.331 (0.805) | 0.978 | | 0.893 (0.101) | 0.904 (0.068) | 0.542 | 3.98 (1.78) | | 4.14 (1.49) | 0.714 |
|  |  |  |  |  | |  |  |  | |  |  |  | |  |  |  |  | |  |  |
|  | 1st | 124.27 (25.65) | 115.41 (27.81) | 0.467 | | 117.19 (24.61) | 109.67 (25.21) | 0.499 | | 4.64 (0.697) | 4.808 (0.307) | 0.353 | | 0.921 (0.055) | 0.943 (0.009) | 0.060 | 3.88 (2.065) | | 3.71 (1.89) | 0.845 |
|  |  |  |  |  | |  |  |  | |  |  |  | |  |  |  |  | |  |  |
|  | 2nd | 103.99 (33.72) | 102.96 (24.65) | 0.929 | | 98.53 (28.76) | 99.38 (24.32) | 0.938 | | 4.6  (0.396) | 4.389 (0.672) | 0.451 | | 0.928 (0.041) | 0.906 (0.067) | 0.434 | 4.15 (1.46) | | 4.42 (1.81) | 0.677 |
|  |  |  |  |  | |  |  |  | |  |  |  | |  |  |  |  | |  |  |
|  | 3rd | 73.97 (24.16) | 74.20 (25.85) | 0.984 | | 72.10 (23.10) | 72.88 (25.58) | 0.944 | | 3.68 (0.966) | 3.795 (1.000) | 0.802 | | 0.820 (0.150) | 0.864 (0.085) | 0.341 | 3.91 (1.82) | | 4.29 (0.488) | 0.598 |
|  |  |  |  |  | |  |  |  | |  |  |  | |  |  |  |  | |  |  |
| Gestational Diabetes  Yes: n = 4  No: n = 32 | Overall | 91.49 (25.54) | 101.99 (34.25) | 0.125 | | 88.56 (31.20) | 97.17 (23.62) | 0.161 | | 4.33 (0.823) | 4.347 (0.669) | 0.932 | | 0.909 (0.099) | 0.893 (0.040) | 0.424 | 4.4 (1.69) | | 3.97 (2.00) | 0.460 |
|  |  |  |  |  | |  |  |  | |  |  |  | |  |  |  |  | |  |  |
|  | 1st | 128.32 (28.93) | 119.75 (22.31) | **0.045** | | 120.56 (26.23) | 107.10 (17.88) | 0.056 | | 4.68 (0.612) | 4.482 (0.809) | 0.784 | | 0.924 (0.051) | 0.922 (0.054) | 0.659 | 3.96 (1.80) | | 3.25 (0.957) | 0.875 |
|  |  |  |  |  | |  |  |  | |  |  |  | |  |  |  |  | |  |  |
|  | 2nd | 77.84 (21.46) | 86.60 (19.74) | 0.165 | | 75.87 (19.98) | 83.70 (20.36) | 0.139 | | 4.03 (0.819) | 4.367 (0.493) | 0.285 | | 0.866 (0.126) | 0.915 (0.036) | 0.440 | 3.97 (1.61) | | 2.00 (1.73) | 0.643 |
|  |  |  |  |  | |  |  |  | |  |  |  | |  |  |  |  | |  |  |
|  | 3rd | 93.55 (30.69) | 129.94 (15.24) | 0.152 | | 90.085 (28.80) | 126.53 (15.53) | 0.129 | | 4.18 (0.896) | 5.282 (0.004) | **0.011** | | 0.8855 (0.095) | 0.957 (0.004) | **0.011** | 4.27 (1.69) | | 6 (1) | 0.457 |
|  |  |  |  |  | |  |  |  | |  |  |  | |  |  |  |  | |  |  |
| Gestational Weight Gain Yes: n = 10  No: n = 26 | Overall | 98.21 (35.57) | 111.55 (32.00) | 0.106 | | 93.49 (32.06) | 106.49 (30.07) | 0.080 | | 4.21 (0.7912) | 4.62 (0.937) | **0.018** | | 0.881 (0.099) | 0.923 (0.100) | **0.025** | 3.92 (1.73) | | 4.79 (1.58) | **0.046** |
|  |  |  |  |  | |  |  |  | |  |  |  | |  |  |  |  | |  |  |
|  | 1st | 136.34 (22.68) | 94.71 (33.14) | 0.341 | | 127.62 (21.77) | 90.36 (29.45) | 0.307 | | 4.61 (0.714) | 4.226 (1.039) | 0.190 | | 0.916 (0.0612) | 0.878 (0.137) | 0.160 | 4.12 (1.97) | | 4.125 (2.1) | 0.486 |
|  |  |  |  |  | |  |  |  | |  |  |  | |  |  |  |  | |  |  |
|  | 2nd | 105.29 (29.88) | 115.86 (31.14) | 0.488 | | 92.22 (25.94) | 103.91 (30.75) | 0.432 | | 4.59 (0.375) | 4.456 (0.752) | 0.101 | | 0.928 (0.022) | 0.907 (0.068) | 0.069 | 4.10 (1.52) | | 4.57 (1.72) | **0.009** |
|  |  |  |  |  | |  |  |  | |  |  |  | |  |  |  |  | |  |  |
|  | 3rd | 75.06 (23.10) | 80.54 (29.99) | **0.040** | | 73.09 (22.06) | 78.69 (28.81) | **0.039** | | 3.75 (0.909) | 3.935 (1.010) | **0.046** | | 0.829 (0.146) | 0.874 (0.084) | 0.074 | 3.75 (1.77) | | 4.13 (0.64) | 0.504 |
|  |  |  |  |  | |  |  |  | |  |  |  | |  |  |  |  | |  |  |

Supplementary Material Table 1: Mean values [with (Standard Deviation) below] of alpha diversity and aMED scores were compared among those who did and did not develop the most common pregnancy complications in the cohort. Pregnancy outcome and microbiome data was available for 36 participants.
