# Supplementary figures and images for: Adherence to Mediterranean diet impacts gastrointestinal microbial diversity throughout pregnancy
Source: BMC Pregnancy Childbirth. 2021 Aug 16;21:558. doi: 10.1186/s12884-021-04033-8 (PMC8369757; doi:10.1186/s12884-021-04033-8)

**Distribution of aMED Scores**

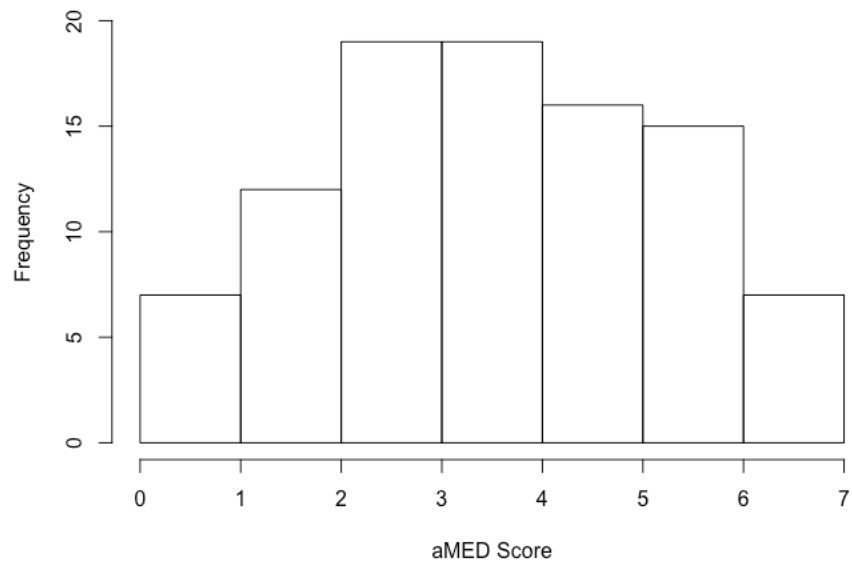

Supplement: Supplementary file 2 — Additional file 2: Fig. S1. Aggregate distribution of all reported energy adjusted aMED scores. [file 12884_2021_4033_MOESM2_ESM.pdf]

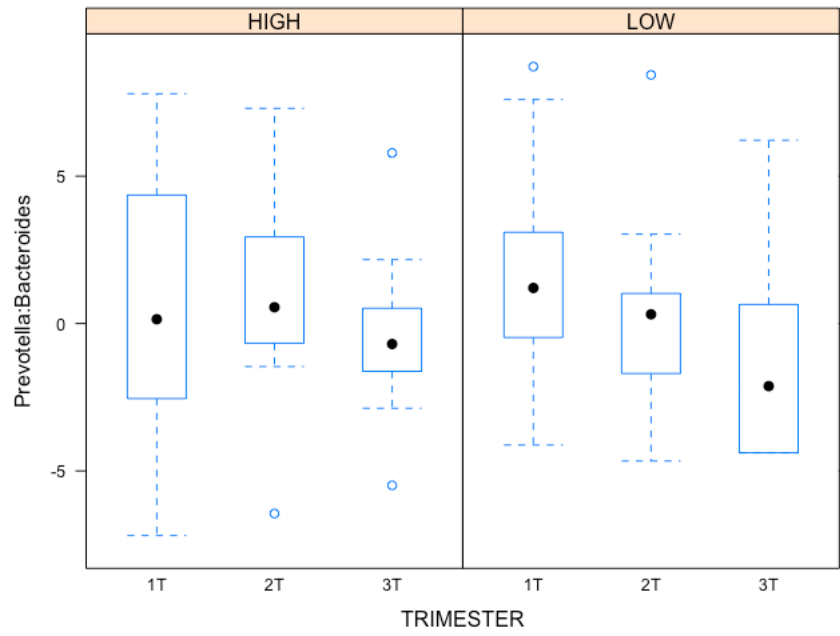

Supplement: Supplementary file 3 — Additional file 3: Fig. S2.Prevotella:Bacteroides Ratios among those above (right panel) and below (left panel) the median aMED score. Participants with lower aMED scores had lower ratios in the third trimester. [file 12884_2021_4033_MOESM3_ESM.pdf]
